# One-Pot Synthesis of Gelatin/Gum Arabic Hydrogels Embedding Silver Nanoparticles as Antibacterial Materials

**DOI:** 10.3390/gels11060429

**Published:** 2025-06-03

**Authors:** Irina Popescu, Irina Mihaela Pelin, Irina Rosca, Marieta Constantin

**Affiliations:** “Petru Poni” Institute of Macromolecular Chemistry, Grigore Ghica Voda Alley 41A, 700487 Iasi, Romania; ipopescu@icmpp.ro (I.P.); rosca.irina@icmpp.ro (I.R.)

**Keywords:** oxidized gum arabic, silver nanoparticles, gelatin, Schiff base reaction, antibacterial, antioxidant

## Abstract

High and large-spectrum antibacterial features and ROS scavenging properties are the most important requirements for efficient wound-dressing materials. A composite hydrogel was synthesized herein by a one-pot procedure embedding silver nanoparticles (AgNPs) covered with oxidized gum arabic (OGA) within gelatin (Gel) hydrogel. Small (2–20 nm), round-shaped AgNPs (ζ = −22 mV) were first obtained by green synthesis using OGA as a reducing and capping agent. Composite hydrogels, containing 0.6 and 1.3 wt.% Ag, were obtained by the covalent cross-linking (Schiff base reaction) of amine groups in gelatin with the dialdehyde groups located on the shell of the AgNPs. Thus, the uniform distribution of the AgNPs in the network contributed to the increased physicochemical and hydrolytic stability of the hydrogels. Moreover, the high swelling degree together with the good mechanical properties make them appropriate candidates for wound-healing materials. The hydrogels exhibited 80% scavenging activity of ABTS^●+^ free radicals after 6 h of incubation and were effective against *E. coli* and *S. aureus,* achieving a 4% survival of bacteria within 3 h (*E. coli*) and 24 h (*S. aureus*). These results clearly indicate that the proposed hydrogels have potential in wound-dressing applications.

## 1. Introduction

Hydrogels based on biomacromolecules have drawn attention in the field of biomedical applications due to their biocompatibility and ability to absorb high amounts of water or biological fluids without their dissolution [1]. Among them, gelatin-based hydrogels have broad applications in the tissue engineering and delivery of therapeutic agents, but also as wound dressings [1,2,3]. Gelatin is a protein obtained from the hydrolysis of collagen that has the advantage of containing the RGD (arginine–glycine–aspartic acid) amino acid sequence required for integrin mediated cell adhesion in the view of tissue formation and remodeling [1,2]. Another advantage of gelatin is its ability to form thermo-reversible gels due to the helix–coil transition [4]; however, hydrogels obtained through the physical cross-linking of gelatin have poor mechanical properties and are not stable for applications under physiological conditions (37 °C) [5]. In this respect, in order to obtain stable hydrogels, chemical cross-linking of gelatin is preferred, which is usually achieved by enzymatic cross-linking, radiation cross-linking, and by its derivatization with radically polymerizable functional groups, or by using different low molecular cross-linkers (i.e., glutaraldehyde, diisocyanates) or coupling agents (carbodiimide). Nevertheless, all these methods present some degrees of toxicity [1,2]. It has been demonstrated that partially oxidized polysaccharides can be used as macromolecular cross-linkers for gelatin, resulting in cyto-compatible hydrogels/scaffolds being obtained [6,7]. Oxidized dextran, pullulan, alginate, xanthan, gum arabic, and other oxidized polysaccharides were used to obtain hybrid hydrogels through a Schiff base reaction between aldehyde groups from polysaccharide and amine groups from gelatin [6,7,8,9,10].

Gum arabic (*Acacia Senegal*) (GA) is a water-soluble gum with a branched structure and low solution viscosity, known for its adhesion, emulsification, and thickening properties [11]. The main chain is mainly composed of 1-3-linked-D-galactopyranosyl units, most of them being branched to C6 atoms. The branches contain galactose units, as in the main chain, but they also contain arabinose (24–27%), rhamnose (12–16%), glucuronic acid (15–16%), and proteins (3%) [12,13]. Apart from having applications in the food industry, GA also has interesting biological activities [13], including anti-inflammatory, antibacterial, and hemostatic effects [11,14,15]. As a result, GA has been used as component in hydrogels with applications in wound dressings [16,17,18,19]. Moreover, GA can be oxidized to obtain dialdehyde-reactive groups for cross-linking with chitosan or collagen/gelatin [6,20,21,22,23].

Hydrogels used as wound dressings should absorb wound exudate, ensure moisture control and mechanical stability, allow gas transmission, have low adherence to the skin, and be biocompatible, biodegradable, and prevent infection [24]. The incorporation of active ingredients that assure the antimicrobial, antioxidant, or anti-inflammatory activities of dressings is also important, especially in the case of chronic wounds [25]. Silver nanoparticles (AgNPs), due to their broad-spectrum antimicrobial activity [26,27], can be entrapped in wound dressings and, because of their ability to sustain the long-term release of silver ions, they do not favor bacterial growth and they promote wound healing [28].

Various natural products, such as plant extracts, amino acids, proteins, or polysaccharides, can be used as reducing agents in the green synthesis of AgNPs [29,30]. Moreover, oxidized polysaccharides, having dialdehyde groups, have been shown to be effective reducing agents [31,32,33]. GA was utilized, alone or in combination with other plant extracts or NaBH_4_ [34], both as a reducing and as a capping agent in the successful synthesis of AgNPs [29,30,35], characterized by round shape and small sizes. However, oxidized GA (OGA) has not yet been utilized for the green synthesis of AgNPs, and it is anticipated that the resulting silver nanogels (OGA-AgNPs) would possess a shell with aldehyde functionality to facilitate covalent bonding with complementary functional groups. In this respect, OGA-AgNPs could be incorporated into gelatin-based hydrogels.

The remaining aldehyde groups from OGA-AgNPs can be involved in the Schiff base formation, contributing to the stabilization of the nanoparticles into the hydrogel. This ex situ approach, where preformed AgNPs are mixed into the polymer solution, followed by cross-linking, would enable the uniform distribution of metallic nanoparticles in the hydrogel. Furthermore, incorporating AgNPs into hydrogels made from natural polymers remains a promising alternative for the long-term release of silver, preventing the agglomeration and accumulation of nanoparticles, and thereby reducing their cytotoxicity [36,37]. The cytotoxicity of AgNPs is an important issue when discussing materials for wound treatment, as local and systemic absorption of silver may occur after prolonged use of wound dressings, leading to elevated levels of silver being detected in blood and urine. Additionally, silver deposition may occur in the lesion and in some organs of the body [38].

**Scheme 1:**
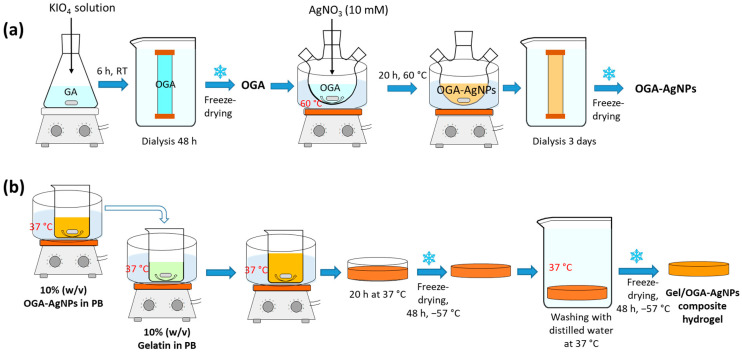
Schematic illustration of the OGA-AgNPs synthesis (**a**) and one-pot preparation process of the composite Gel/OGA-AgNPs hydrogels (**b**).

Therefore, the objective of this work was to synthesize composite hydrogels through the Schiff base reaction between gelatin and OGA, incorporating AgNPs (Gel/OGA-AgNPs). First, AgNPs were obtained in the presence of OGA as a reducing agent and stabilizer and further characterized. Gelatin/OGA hydrogels without AgNPs were obtained to establish the most appropriate ratio between the biopolymers; then, composite materials embedding AgNPs were synthesized by a one-pot procedure in which half or all the amount of OGA was replaced with OGA-AgNPs. The preparation process of the composite Gel/OGA-AgNPs hydrogels is shown in Scheme 1. The physicochemical properties of the hydrogels were studied, including gel fraction, cross-linking density, porosity, swelling in physiological conditions, mechanical properties, and hydrolytic degradation. The biological properties, like antioxidant capacity and antibacterial activity against *S. aureus* and *E. coli*, were also assessed. The antibacterial hydrogels synthesized in this study could have potential applications as wound dressings.

## 2. Results and Discussion

### 2.1. Oxidation of GA

It is well known that GA, containing vicinal hydroxyl groups on the polysaccharide chain, can be oxidized by periodate to introduce aldehyde groups [6,23,39]. A recent paper showed that oxidation occurs mainly at the terminal rhamnose and arabinose subunits of the branched polymer [40]. Therefore, in the present work, GA was oxidized using KIO_4_ (10:4 molar ratio between the saccharide unit of GA and KIO_4_), with the oxidized product having 2.93 meq/g of aldehyde groups, corresponding to an oxidation degree of 25.8%. The average molecular weight of OGA was determined by viscometry as 467 kDa, while that of GA was 493 kDa, indicating that only a very slight degradation of the molecular mass had occurred during the oxidation. The oxidation of linear polysaccharides usually takes place with the backbone cleavage [41], but in the case of branched GA, this cleavage does not produce a drastic decrease in the molecular mass [6].

**Figure 1:**
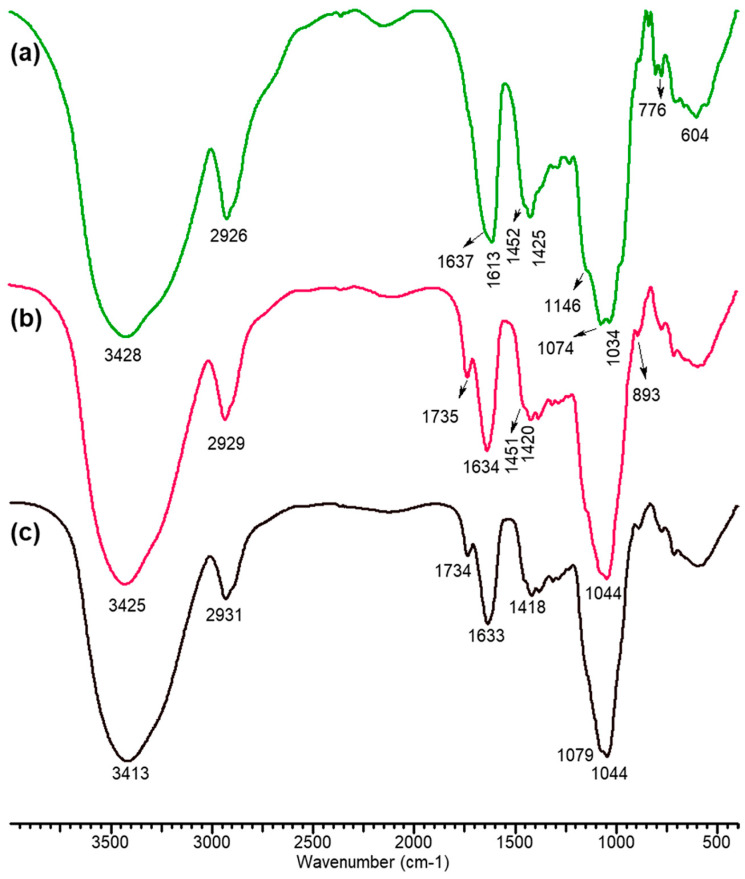
FT-IR spectra of GA (**a**), OGA (**b**), and OGA-AgNPs (**c**).

The proof of the oxidation reaction was emphasized by comparing the FT-IR and ^1^H-NMR spectra of the native GA and OGA (Figure 1 and Figure S1, respectively). As shown in Figure 1, in the FTIR spectra of OGA, all the characteristic bands from GA are present: between 3000 and 3600 cm^−1^ (O-H stretching), 2929 cm^−1^ (symmetric and asymmetric aliphatic C-H and -CH_2_ stretching vibrations from sugars, galactose, arabinose, and rhamnose), 1637 and 1613 cm^−1^ (carboxylic groups from glucuronic acid, and amide N-H bend from galactoproteins), 1425 cm^−1^ (the O-H bending of carboxylic group), and between 850 and 1200 cm^−1^ (C-O, C-C and C-O-C stretching, C-O-H bending) [39,42,43]. The oxidation of the polysaccharide is demonstrated by the appearance of new bands at 1735 cm^−1^, attributed to the vibration of the aldehydic carbonyl groups, and at 893 cm^−1^, corresponding to the stretching vibration of hemiacetal structures [23,33,40]. The proton spectrum of GA (Figure S1) agrees with those reported in literature by Nie et al. [44]. As seen in the ^1^H-NMR spectrum of OGA (Figure S1), new signals appeared at 9.56 ppm and 8.32 ppm, which are assigned to aldehydic groups and formic acid (secondary product in the oxidation reaction), respectively. The small intensity of peak at 9.56 ppm, along with the changes in the intensity of some signals (Figure S1), may indicate the presence of hemiacetals, as evidenced in the FT-IR spectrum. Also, the decrease in intensity of the signals at 1.25 ppm (methyl protons in rhamnopyranoside) and the appearance of two new signals at 1.17 and 1.11 ppm (new methyl protons from oxidized rhamnopyranoside) represent evidence that the terminal rhamnopyranoside and arabinofuranoside units are oxidized whereas the (in-chain) arabinofuranoside and galacturonic acid are unaffected [40]. The two spectroscopic methods confirmed the outcome of the oxidation reaction of GA.

These new aldehyde groups from OGA can be further used as reducing moieties for obtaining AgNPs, and they can also form Schiff base bonds with amine groups from gelatin to create cross-linked hydrogels.

### 2.2. Synthesis and Characterization of OGA-AgNPs

**Figure 2:**
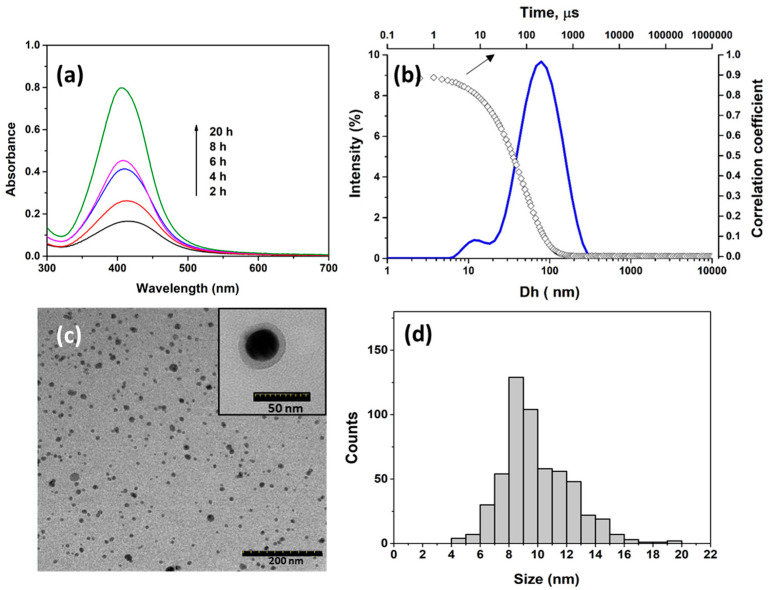
(**a**) UV–Vis spectra of AgNPs prepared using 1 wt.% aqueous solution of OGA (pH = 7 and 60 °C) at different reaction times. (**b**) Size distribution of OGA-AgNPs in aqueous solution measured by DLS. (**c**) TEM images and (**d**) the size histogram of the nanoparticles obtained after 20 h reaction time.

The reduction of silver ions to metallic silver can be performed both by the aldehyde groups resulting from the oxidation of GA and by the primary alcohol groups (Scheme 1a). To accelerate this process, an increased temperature was used (60 °C). The formation of AgNPs can be monitored by UV–Vis spectroscopy due to the surface plasmon resonance properties of the nanoparticles. The increase in the nanoparticles’ size is accompanied by an increase in the absorption wavelength (higher than 400 nm) [45]. Moreover, as a result, the increase in the nanoparticles’ concentrations has higher values of absorbance. Figure 2a shows the increase in UV absorption with reaction time, but we can also see a small blue shift from 415 nm to 405 nm. These results indicate that the number of AgNPs increases in time, but their size decreases due to the nucleation of new nanoparticles, and the adsorption of OGA onto the surface of already formed nanoparticles limits their growth. High yield and small size of AgNPs were obtained after 20 h, so these nanoparticles with absorption bands centered at 405 nm were used in further experiments. It is known that plasmon bands located at around 400 nm correspond to small spherical AgNPs [45,46]. The dimensional distribution of OGA-AgNPs in aqueous solution (Figure 2b) determined by DLS was bimodal with a polydispersity index of 0.285. The average size was 58.6 ± 0.8 nm, lower than that of AgNPs covered with GA [35] or other linear polysaccharides like chitosan [47] or oxidized pullulan [33]. The branched OGA from the surface of the metallic nanoparticles has a stabilization effect due to the carboxylic groups, a fact demonstrated by the negative zeta potential (−20.9 ± 0.2 mV). The spherical shape of the metallic core of OGA-AgNPs can be observed in TEM images (Figure 2c). The size of the AgNPs ranged between 4 and 20 nm (Figure 2d); these values are close to the size of the AgNPs obtained in the presence of GA [29,30,31]. The organic polysaccharide acts as capping agent and can be seen on the surface of the nanoparticles (Figure 2c, inset).

As seen in the FTIR spectrum of the OGA-AgNPs (Figure 1c) upon the reduction of silver nitrate by OGA, the intensity of the band from 1735 cm^−1^, characteristic of CH=O groups, is reduced. This demonstrates the participation of the aldehyde groups in the reduction of silver ions, as well as the fact that some of the aldehyde groups remained available for further reactions. The FTIR spectrum of OGA-AgNPs also shows a shifting of 3413 cm^−1^, 1633 cm^−1^, and 1418 cm^−1^ bands corresponding to –OH, –C=O, and O-H vibrations in carboxylic groups, respectively, demonstrating their contribution to the reduction of Ag^+^ ions and the stabilization of OGA-AgNPs. The content of aldehyde groups in the OGA-AgNPs was determined by the titration method with hydroxylamine, and was found to be 1.96 meq/g, which means that 66% of the initial groups in OGA are involved in the formation and the stabilization of AgNPs.

As determined by atomic absorption spectroscopy (AAS), the obtained OGA-AgNPs contain 3.06 ± 0.02% silver, meaning that 96.94% by weight is represented by a shell of the polysaccharide (OGA).

### 2.3. Preparation and Characterization of Hydrogels with and Without AgNPs

Gelatin/OGA (Gel/OGA) hydrogels without or with AgNPs were prepared based on the cross-linking reaction between the amine groups from gelatin and the aldehyde groups of branched OGA or OGA-AgNPs. The cross-linking reactions were performed in phosphate buffer at pH = 7.4 and 37 °C when Gel did not form physical gels. In fact, Gel in solution at 37 °C has high chain mobility and, at physiological pH, the amine groups are not protonated; therefore, the probability of Schiff base formation with the aldehyde groups of OGA is highest [48]. To find the optimum composition, hydrogels without AgNPs were first prepared by varying the gravimetric ratio between Gel and OGA between 40:60 and 60:40 (samples H1, H2, and H3 in Table 1). The hydrogels were washed with water at 37 °C to remove the unreacted polymers. It should be mentioned that, when one of the components was in a much higher mass ratio (25:75 or 75:25), the obtained hydrogels were highly swollen and lost their integrity.

The theoretical molar ratio between the amine groups from Gel and aldehyde groups from OGA could be calculated (see Table 1) knowing the content of aldehyde groups from OGA and OGA-AgNPs, and by determining the content of amine groups in Gel as (33.9 ± 0.2) × 10^−5^ moles/g; this is a value comparable to the data reported in the literature [49].

The gel fraction (GF,%), an important parameter when preparing hydrogels, represents the percentage of the cross-linked macromolecules that remained after the hydrogel purification. As shown in Table 1, the GF was around 86.5%, and slightly increased with the increase in the aldehyde/amine ratio (the increase in OGA content). Since gelatin can be easily solubilized in an aqueous environment at 37 °C, it can be concluded that the high values of GF are provided by the Schiff base cross-linking. The degree of covalent cross-linking was determined by comparing the content of the free amine groups in hydrogels before washing with the content of the theoretical amine groups of Gel in the initial mixture. It was found that the cross-linking of NH_2_ groups increased from 55.9% to 70% by increasing the molar ratio between the reactive groups of OGA and Gel (Table 1). Similar degrees of cross-linking were reported for other gelatin-based hydrogels obtained via Schiff base reaction with oxidized polysaccharides [6,7,9]. Our values are slightly higher than those found for gelatin/OGA [6] due to the higher oxidation degree of OGA. Also, the higher ratio of the used OGA enabled a better cross-linking efficiency to be achieved by comparison with gelatin/oxidized pullulan hydrogels [7], dialdehyde sodium alginate [9], or dextran [9].

**Figure 3:**
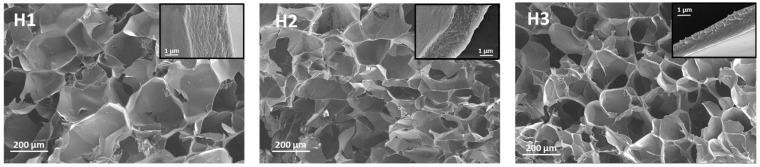
Scanning electron micrographs of the H1, H2, and H3 hydrogels together with the cross-section of the pore walls (inset).

The morphology and porosity of hydrogels were influenced by the ratio between the reactive groups of polymers (Figure 3), a more uniform network being observed at the highest CHO:NH_2_ molar ratio (H3 sample). All samples have well-defined pores with thick walls (1.5–3.3 µm). The sample H1, with the lowest cross-linking degree, has the largest pores (120 ± 35 µm) but the thickest pore walls (Figure 3, insets). With the increase in the cross-linking degree, the pores’ size decreased to 92 ± 29 μm for H2 sample and to 84 ± 26 μm for H3 hydrogel, respectively; however, no remarkable differences were observed between samples H2 and H3.

The average porosity of the hydrogels (P, %) increased from 70 to 77% with the increase in OGA content (Table 1). The lowest porosity characterizing the H1 sample can be explained by considering the thicker pore walls observed for this hydrogel in SEM images.

The hydrogels’ swelling capacity was studied using simulated skin physiological conditions (phosphate buffer pH = 5.5 and 37 °C), and they proved to have a relatively fast swelling, reaching equilibrium in the first hour due to their high porosity. The swelling ratios (SRs) at equilibrium, presented in Table 1, are situated in the range 7.2–9.1 g/g, increasing from sample H1 to H3, due to the increase in the hydrogel porosity in this order. Similarly, Sharika et al. [6] found SR values of 66 and 56% for gelatin/OGA hydrogels cross-linked in the presence of borax, which increased the cross-linking degree of the network. The SRs were also higher than the values found by Wang et al. [9] for gelatin/oxidized guar gum and gelatin/oxidized dextran hydrogels; the higher cross-linking degree of these hydrogels induced by the higher oxidation degree of polysaccharides influenced the porosity and hence the swelling capacity. The degree of swelling in simulated pH found in the infected wounds (pH = 7.4) [50] follows the same pattern as at pH 5.5, with values slightly lower than those at pH = 5.5, due to an increase in the degree of cross-linking (fewer free NH_2_ groups in Gel) on one side, and an increase in the OGA content on the other side. It can also be considered that the number of anionic carboxylic groups increases with the rise in OGA: Gel ratio, exceeding the number of free amine groups and increasing the ionic swelling pressure in the hydrogel. These SR values suggest that the Gel/OGA hydrogels have a high swelling capability, making them suitable for wound-healing applications [51].

**Figure 4:**
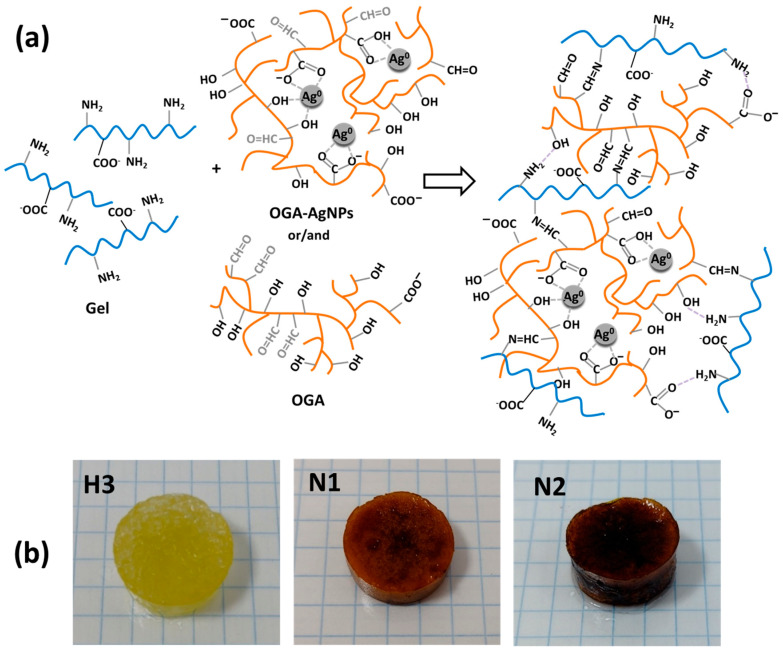
(**a**) Schematic representation of the synthesis of composite hydrogels Gel/OGA-AgNPs and (**b**) optical images of hydrogels without (H3) and with AgNPs (N1 and N2).

The H3 hydrogel showed the highest cross-linking density and gel fraction, with the highest porosity and swelling performances; these properties are required in the field of wound-healing materials. Therefore, the composite hydrogels embedding AgNPs (N1 and N2 samples in Table 1) were obtained starting from the 40:60 gravimetric ratio between Gel and OGA. As mentioned earlier, the preparation method consisted of a single-step procedure involving the mixing of OGA-AgNPs and Gel solutions (Scheme 1b). To incorporate different amounts of AgNPs, the initial OGA amount used in the synthesis of the H3 sample was partially (N1) or totally (N2) replaced with OGA-AgNPs (Table 1). The schematic representation of possible interactions between the polymeric components during the preparation of composite hydrogels is presented in Figure 4a. As can be seen in the optical images of the hydrogels presented in Figure 4b, the introduction of OGA-AgNPs led to a uniform brown coloration of the N1 and N2 samples.

By replacing OGA with OGA-AgNPs, the cross-linking degree of amine groups in Gel decreased as a result of the lower amount of aldehyde groups of OGA covering AgNPs (Table 1). However, no significant decrease in GF values (*p* < 0.05) was observed, which could be due to the favored formation of additional H-bonds between OGA covering AgNPs and amine and hydroxyl groups in Gel. This supplementary physical cross-linking of the composite hydrogels network, possibly induced by the more collapsed chain conformation of OGA covering AgNPs—compared with free OGA in H3 sample—along with the hydrophobic effect of AgNPs [52] conferred a smaller porosity and swelling capacity (Table 1). The amount of Ag in Gel/OGA-AgNPs hydrogels, determined by AAS, showed a slight difference between the theoretical and the practical content, indicating that the OGA-AgNPs were effectively incorporated into the network structure and were not removed during the purification step. The silver content of the composite hydrogels prepared here falls within the accepted values (around 1%) to ensure a balance between cytotoxicity and antibacterial properties [53]. The chemical structure of Gel/OGA-AgNPs hydrogels was confirmed by comparative analysis of the FT-IR spectra of Gel/OGA-AgNPs hydrogel (sample N2), Gel, and OGA-AgNPs (Figure S2). The characteristic bands of the two polymeric components remained in the hydrogel but some differences could be observed. For example, the band at 1640 cm^−1^ sharpened and shifted to lower wavenumbers compared to Gel (1644 cm^−1^) and the absorption band at 1734 cm^−1^ in the OGA-AgNPs spectrum disappeared in hydrogel, indicating the consumption of aldehyde groups and the formation of Schiff base bonds [54]. In the deconvoluted 1500–1745 cm^−1^ region of the N2 spectrum, in addition to the amide I stretching vibration in Gel (1636 cm^−1^), a new band appeared at 1679 cm^−1^ which can be attributed to the newly formed imine bonds (C=N). The stretching vibrations of amide A and amide B in Gel, shifted to low wavenumbers in Gel/OGA-AgNPs hydrogel, indicating strong intermolecular H-bonding between components [55]. At the same time, the amide II (1543 cm^−1^) and amide III (1231 cm^−1^) bands are slightly shifted to 1542 and 1241 cm^−1^, respectively, and the symmetrical vibration of COO^-^ at 1388 cm^−1^ shifted to a low value of 1386 cm^−1^. These results proved the intermolecular interaction between Gel and OGA-AgNPs [56].

**Figure 5:**
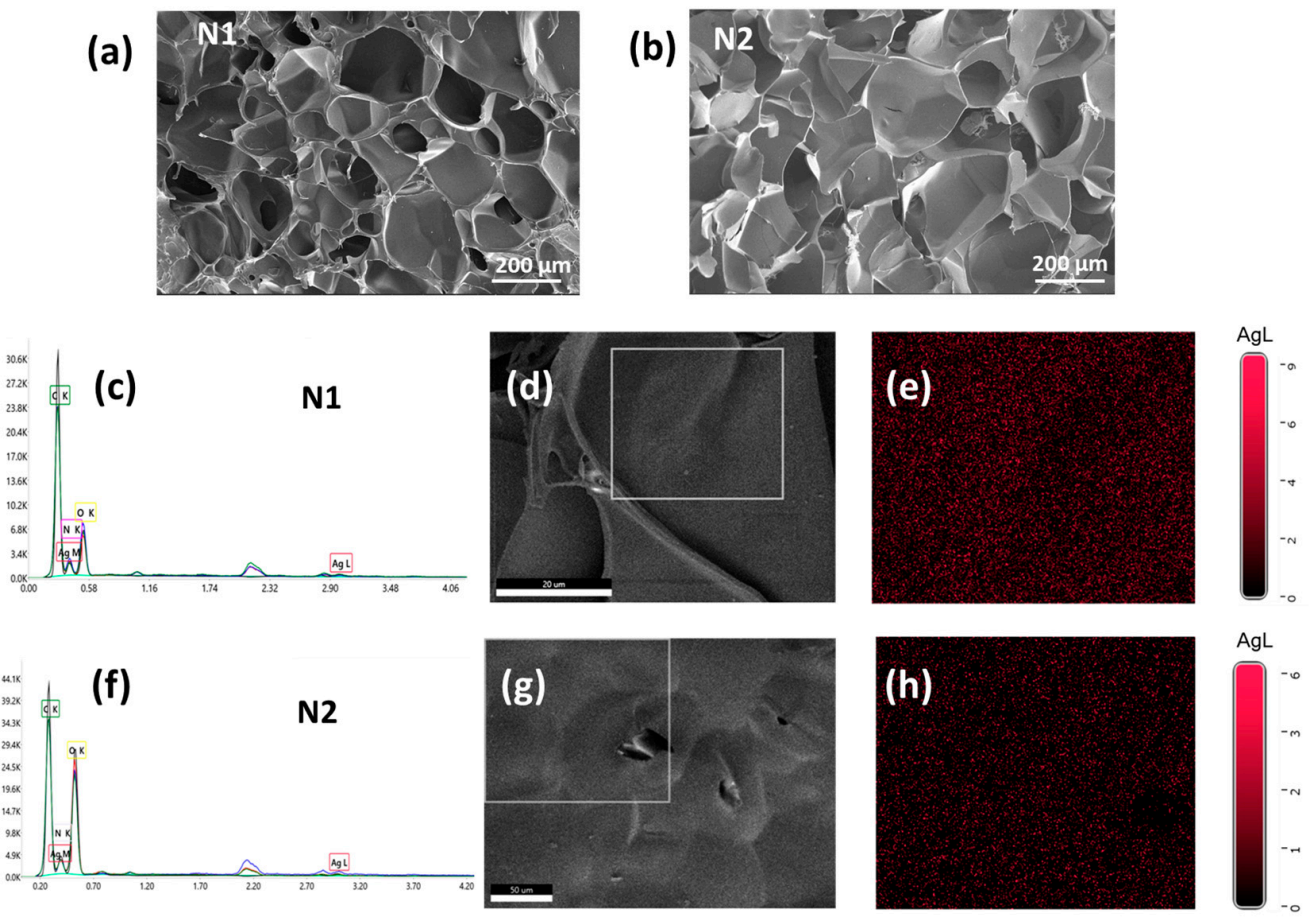
Microphotographs (cross-section) of the N1 (**a**) and N2 (**b**) hydrogels; EDX spectra of the N1 (**c**) and N2 (**f**) hydrogels; SEM image of the mapped zone (**d**,**g**) and mapping images for Ag (**e**,**h**).

The SEM images of the nanocomposite hydrogels (Figure 5a,b) show a porous structure, indicating the successful formation of a uniform Gel/OGA-AgNPs network in the composite hydrogels, with a large distribution of the pores’ diameters, ranging between 40 and 220 nm. The average pore sizes of the hydrogels increased from 96 ± 34 μm to 120 ± 40 μm with higher OGA-AgNPs content, which may be attributed to the formation of more cross-linking centers, leading to a closer aggregation of the OGA shell of AgNPs. The presence of AgNPs in the composite hydrogels after purification was demonstrated by energy-dispersive X-ray (EDX) analysis (Figure 5c,f). As expected, the peaks corresponding to the Ag element are higher in the N2 sample than in the N1 one. The EDX elemental image of Ag (Figure 5e,h) in N2 hydrogel showed that the distribution of AgNPs is uniform. This proves that the ex situ approach used in preparing the composite hydrogels (including the mixture of the preformed OGA-AgNPs in the polymer solution before cross-linking) led to a very uniform distribution of the metallic nanoparticles in the hydrogel matrix, a distribution that cannot be achieved using an in situ method where the AgNPs are generated inside the hydrogel [57,58].

### 2.4. Compression Test

**Figure 6:**
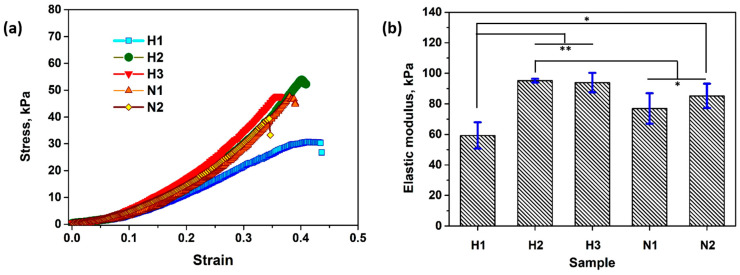
Stress–strain compression curves of the swollen hydrogels (**a**) and the obtained elastic modulus (**b**). Results are expressed as means ± standard deviation (S.D.) of three (n = 3) independent experiments; * *p* < 0.05, ** *p* < 0.01 among the samples.

The mechanical properties of hydrogels are important for their biomedical applications. For that reason, uniaxial compression tests were performed on the swollen hydrogels in simulated skin physiological medium (PB with pH = 5.5), and the stress–strain curves are presented in Figure 6a. The shape of the compression curves and the values of the compression strength (σ) for the Gel/OGA hydrogels are similar to those reported for porous hydrogels from Gel and other oxidized polysaccharides [9,59]. It is known that the mechanical properties depend on the cross-linking density (physical and chemical) and the internal network structure of the hydrogels [60]; thus, the lowest mechanical properties were obtained for the H1 hydrogel (E = 59 kPa and σ = 29 kPa) with the lowest cross-linking degree, while hydrogels H2 and H3 displayed higher elastic modulus (~94 kPa) and compressive strength (~50 kPa). The behaviors of H2 and H3 at compression ae similar (*p* < 0.05) due to their similarity in cross-linking degree, porosity, and swelling capacity (see Table 1).

By adding AgNPs, the mechanical stability of the composite hydrogels slightly decreased by comparison with the empty hydrogel (H3 sample) (Figure 6a,b), mainly due to the rigidity brought by the metallic nanoparticles and the lower covalent cross-linking degree of these samples (see Table 1). However, increasing the amount of AgNPs from 0.6% to 1.3% by the partial or total replacement of OGA with OGA-AgNPs in N1 and N2 samples has led to an augmentation of E value from 77 to 85 kPa, close to that of the empty hydrogel. The same behavior was observed when chitosan–AgNPs were introduced into hydrogels based on PVA/chitosan/oxalic acid [53]. The relatively low values for the Young’s modulus for all the samples (60–95 kPa) fits to the elasticity requirements in wound-dressing applications [61].

The compressive modulus values of Gel/OGA-AgNPs hydrogels are in a suitable range compared to the various types of hydrogels already published. These include composite hydrogels obtained from gelatin with other oxidized polysaccharides, such as oxidized gum arabic and acrylamide [55], oxidized sodium alginate [62], and oxidized chondroitin sulfate [63]; in addition, these include other polysaccharide-based hydrogels, for example, carboxyethyl chitosan and oxidized sodium alginate containing tannic acid/red cabbage functionalized silver nanoparticles [64], or hydrogels containing 30% commercial block copolymer poly(ethylene oxide)-poly(propylene oxide)-poly(ethylene oxide) F 127 and 0.5% alginate [65]. For hydrogels based on gelatin and dialdehyde derivatives of sodium alginate, guar gum, and dextran, Wang et al. [9] reported a lower Young’s modulus than the hydrogels synthesized here (around 0.2 kPa). This discrepancy is due to the different methods used to prepare the hydrogels; in our case, the compression test was performed on fully rehydrated hydrogels after the purification and lyophilization steps.

### 2.5. Hydrolytic Stability

**Figure 7:**
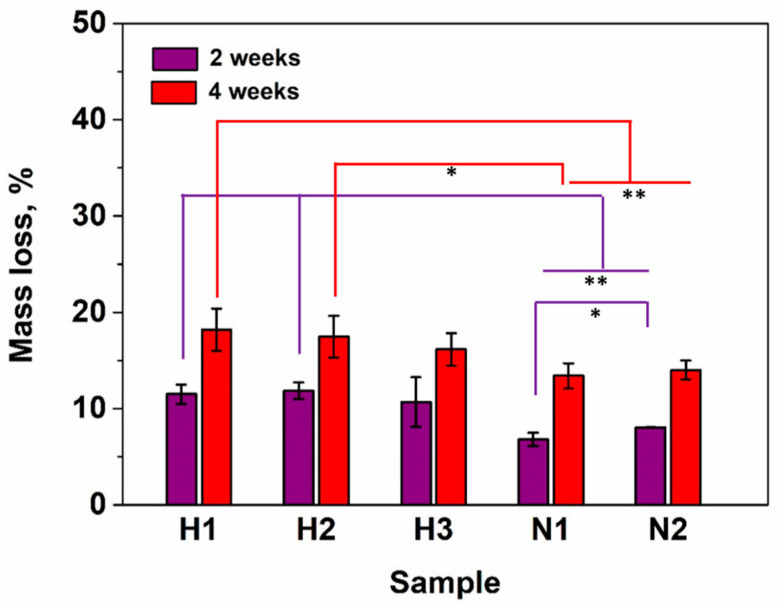
Percentage of weight loss (%) after 2 and 4 weeks of incubation in PB (pH = 5.5) at 37 °C of hydrogels without and with AgNPs. Results are expressed as means ± standard deviation (S.D.) of three (n = 3) independent experiments; * *p* < 0.05, ** *p* < 0.01 among the samples.

The stability of hydrogel composites in physiological conditions represents an imperative feature for biological applications. Therefore, in vitro degradation was evaluated by incubating the hydrogels in PB (pH = 5.5) at 37 °C for 2 and 4 weeks, and the results are presented in Figure 7. All the hydrogels have a low degradation rate, with less than 20% hydrogel mass loss after 4 weeks. In general, the hydrogels obtained by the cross-linking of gelatin have relatively good hydrolytic stability, since the chemical attack of water molecules on amide bonds from the gelatin backbone takes place at low rates at neutral pH [5,66]. The hydrolytic degradation of Schiff bases also has low-rate constants in the neutral pH range, including pH = 5.5 [67], explaining the relatively low degradation of Schiff base cross-linked Gel/OGA hydrogels. Such a good stability to hydrolytic degradation was found for other chemically cross-linked gelatin hydrogels [5,6,66].

The degradation rate of hydrogels is known to decrease with the increase in the cross-linking density [59,68], so the samples H2 and H3 have slightly lower weight loss compared with H1 sample. The composite hydrogels have better hydrolytic stability than those without AgNPs, most probably due to the hydrophobicity induced by the AgNPs.

As a result, it can be concluded that the chosen preparation approach of Gel/OGA-AgNPs hydrogels through ex situ synthesis of AgNPs covered by OGA and one-pot synthesis of hydrogels using OGA-AgNPs as one of the polymeric components confers good stability on the composite gelatin/OGA hydrogel in physiological conditions.

### 2.6. Biological Activity of Composite Hydrogels

#### 2.6.1. In Vitro Evaluation of Antioxidant Activity

Wound dressings with antioxidant activity can control the levels of reactive oxygen species (ROS) and oxidative stress, accelerating wound healing [69]. It is known that radical species can induce the oxidation of different amino acids (cysteine, tryptophan, tyrosine, phenylalanine, histidine) from the structure of the proteins [70]. Thus, proteins like gelatin, but especially the peptides obtained from its hydrolysis, exhibit antioxidant activity [70,71]. The antioxidant activity of AgNPs was also studied in the literature [72], and green-synthesized AgNPs obtained using plant extracts were proven to inhibit free radicals due to the presence of phenolic compounds [72]. Still, AgNPs obtained in the presence of exopolysaccharides [73,74], glycoproteins [75], or polysaccharide gums like galactomannan or κ-carrageenan [76] are also found to have antioxidant activity. This can be explained by the reduction power of the AgNPs [74], or the catalytic activity of AgNPs in the redox reactions [77] between amino acids and the free radicals.

**Figure 8:**
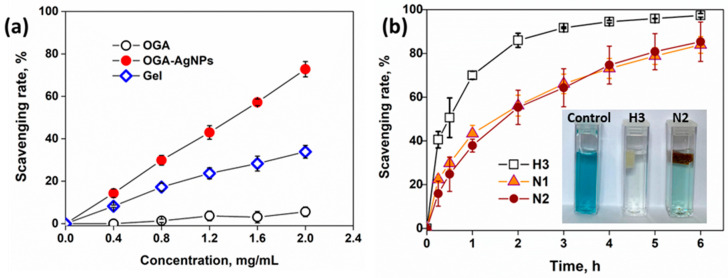
ABTS^●+^ radical scavenging activity of different components of the hydrogels in solution (measured after 2 h) (**a**) and of the hydrogels without and with AgNPs measured in time (**b**). Photographs of the ABTS solution in the absence and presence of H3 and N2 hydrogels after 5 h of immersion (inset).

The antioxidant activity of the composite hydrogel precursors (OGA, OGA-AgNPs, and Gel) was first studied in aqueous solution using 2,2′-azino-bis-(3-ethyl)benzothiazoline-6-sulfonic acid cation radical (ABTS^●+^). As can be observed from Figure 8a, gelatin and OGA-AgNPs have radical scavenging activity in a concentration-dependent manner, while OGA does not show noticeable antioxidant activity. At a relatively high concentration—such as 2 mg/mL—OGA-AgNPs inhibit 73% of the ABTS^●+^ radicals, while Gel inhibits only 34%. The higher antioxidant activity of OGA-AgNPs compared with that of the oxidized polysaccharide can be explained by their smaller size and large specific surface area, contributing to more free radical scavengers and better antioxidant activity [78]. Nevertheless, the IC_50_ value (concentration providing inhibition of 50% of radicals) of OGA-AgNPs was 1.4 mg/mL, much higher than the value for ascorbic acid (0.045 mg/mL), meaning that the antioxidant activity of the composite nanoparticles was relatively low.

The scavenging activity of the hydrogels against ABTS^●+^ free radicals was studied over time (Figure 8b). Surprisingly, the H3 hydrogel without AgNPs, showed a high antioxidant activity, inhibiting 90% of the radicals after 2 h. This behavior may result from the synergistic effect of the two polymeric components whose calculated concentration in the amount of the tested hydrogel is 10 times higher than their concentration in the solution. The influence of the radical scavenging activity via Schiff bases’ electron and proton transport mechanisms must also be considered [79,80]. As illustrated in Figure 8b, the composite hydrogels N1 and N2 exhibited lower radical scavenging activity than H3, attributed to their reduced content of Schiff bases and the diminished availability of reactive groups caused by their smaller porosity and swelling capacity (Table 1). The decrease in the Schiff base amount from N1 to N2 hydrogels is compensated by the amount of OGA-AgNPs with antioxidant activity, so after 6 h of incubation, 80% of the ABTS^●+^ are inhibited, in the cases of both N1 and N2. The evidence of the radical scavenging activity of H3 and N2 samples is presented as an inset of Figure 5b, where the discoloration of ABTS solution after 5 h of immersion can be observed.

#### 2.6.2. Antibacterial Studies

The antimicrobial activity of the OGA-AgNPs was studied in solution and the minimum inhibitory concentration (MIC) determined by the serial dilution method was 250 µg/mL for both bacterial strains taken into consideration: *S. aureus* and *E. coli*. This value is relatively high; however, considering the Ag content in the OGA-AgNPs, the MIC corresponds to a concentration of AgNPs of 7.65 µg/mL. This value is in agreement with the MIC found in the literature for AgNPs against *S. aureus* and *E. coli* that ranged in the domains to 6.7–50 µg/mL and 6.25–30 µg/mL, respectively [26,33,81,82,83,84]. Therefore, it can be stated that OGA-AgNPs synthesized here and characterized by spherical shape and sizes between 2 and 20 nm have good antimicrobial activity.

**Figure 9:**
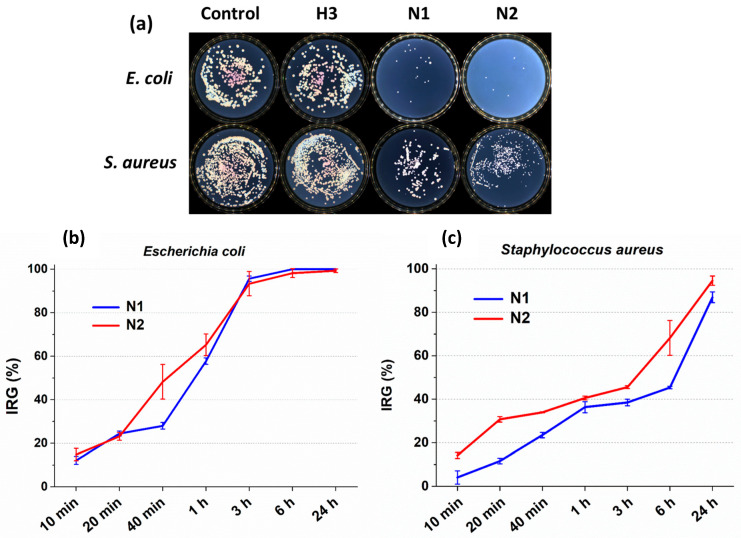
In vitro antibacterial activity of the hydrogels after 3 h of incubation with *E. coli* and *S. aureus* (**a**); growth inhibition of the bacterial cells expressed as a percent inhibition vs. incubation time in the case of samples in contact with *E. coli* (**b**) and *S. aureus* (**c**).

The growth inhibition of the bacterial colonies (*S. aureus* and *E. coli*) in the presence of hydrogels without (H3 sample) and with AgNPs (N1 and N2 samples) was evaluated by the viable cell-counting method [54] (Figure 9). The results showed that H3 did not exhibit antibacterial activity against the tested bacteria (Figure 9a). Depending on the amount of OGA-AgNPs, the composite hydrogels presented high antimicrobial activity against *E. coli*, a Gram-negative bacterial strain, so after 3 h of incubation, the bacterial inhibition was around 96% for both N1 and N2 samples (Figure 9a,b). After 24 h of incubation, no colony-forming units were observed. The antibacterial activity of the composite hydrogels was lower for the Gram-positive bacterial strain represented by *S. aureus*. In this case, the inhibition ratio of the growth of bacteria (IRG) after 3 h of incubation was 38% for N1 sample and 45% for N2 one, respectively (Figure 9a,c). After 24 h, IRG% increased to more than 87% and 95% for N1 and N2, respectively (Figure 9b).

The antibacterial activity of the composite hydrogels relies on the presence of AgNPs, as it is well known that these can destroy both Gram-positive and Gram-negative bacterial cells, including multidrug-resistant bacteria [26] by generating destabilization of the inner membrane, increasing its permeability, and inducing the leakage of the cellular content or the alteration of the metabolic pathways by the released Ag^+^ [85]. The differences between the efficiency of N1 and N2 samples against the two types of bacterial strains tested may be explained by the differences between the two types of cell walls. Gram-positive bacterial cells have a thick peptidoglycan layer (~80 nm) with covalently attached teichoic and teichuronic acids which, due to their negative charge, give them resistance to AgNPs. In contrast, Gram-negative bacterial cells possess a thin peptidoglycan layer (15 nm) and an outer lipopolysaccharide membrane, which facilitate the entry of the Ag ions released from hydrogels into the cell [86]. The antibacterial activity of the composite hydrogels can be related to the following: (i) the direct contact of the bacteria with AgNPs entrapped in hydrogels, followed by the generation of ROS by the nanosilver; (ii) the continuous release of silver ions from AgNPs [87] facilitated by the diffusion of water molecules into hydrogels. The AgNPs released from the swollen hydrogels will be oxidized into silver ions, which will adhere to the cell wall and cytoplasmic membrane and interact electrostatically with the thiol group of amino acids present in the proteins. Once adhered, the inhibition of the respiratory system of bacterial cells will cause their death [88], the disruption and inhibition of DNA replication, and therefore the cells’ multiplication. In addition, silver ions can inhibit protein synthesis by denaturing ribosomes in the cytoplasm [89].

The antibacterial efficacy of Gel/OGA-AgNPs can be mainly associated with the release of Ag^+^ ions from the polymeric network. The release pattern of Ag^+^ ions from N1 and N2 samples in phosphate buffer (Figure S3) showed a two-phase characteristic. The release in the first 6 h was fast due to the diffusion of Ag^+^ ions or AgNPs situated at the surface of hydrogels. Then, the Ag^+^ ions from the inside of the network began to be released at a slower rate. The amount of silver released in the first 6 h was higher from the N2 sample, with more loaded AgNPs, confirming the higher antibacterial effect of this sample (Figure 9). The smaller swelling degree and porosity of this sample (Table 1) restricted the water diffusion and slowed down the Ag^+^ release rate over time. This behavior is in line with other results obtained for Gel/alginate [56] or chitosan/PVA [53] hydrogels containing AgNPs. After 7 days of immersion in PB, the amount of Ag^+^ released was only 2.7% of the total Ag content, indicating that most of the AgNPs remained in the hydrogel. All these results demonstrate a good stability and long-term release of AgNPs incorporated in Gel/OGA-AgNPs hydrogels, properties which may contribute to a decrease in the cytotoxic effect of the materials [38].

Gum arabic has been previously reported to have topical application in skin lesions [16,17,90], and the presence of AgNPs brings additional efficiency in wound healing.

## 3. Conclusions

Porous hydrogels with good hydrolytic stability have been obtained from Gel and OGA by Schiff base cross-linking. The porosity, cross-linking degree, swelling ratio, and mechanical properties of the hydrogels are influenced by the ratio between Gel and the dialdehyde gum arabic. OGA was also used as a reducing and capping agent in the synthesis of small spherical AgNPs with diameters between 2 and 20 nm. The AgNPs covered with OGA retain some aldehyde groups and can act as a cross-linker for Gel. Thus, composite Gel/OGA hydrogels with AgNPs entrapped in the network structure were obtained with a uniform distribution of the metallic nanoparticles. The composite hydrogel containing 0.6% or 1.3% silver showed mechanical properties similar to the hydrogel without AgNPs, improved hydrolytic stability, and most importantly antioxidant and antimicrobial activity. Gram-negative bacteria like *E. coli* were inhibited after 3 h of incubation; meanwhile, in the case of Gram-positive bacteria, like *S. aureus*, the inhibition increased with the rise in the amount of AgNPs in the composite hydrogels. These results indicate that the proposed Gel/OGA-AgNPs hydrogel has potential in wound-dressing applications.

## 4. Materials and Methods

### 4.1. Materials

Gum arabic (GA) from acacia tree, branched polysaccharide, gelatin (Gel) Type B from bovine skin (gel strength ~225 g Bloom), potassium periodate (KIO_4_), 2,2′-Azino-bis-(3-ethyl)benzothiazoline-6-sulfonic acid (ABTS), and hydrindantin dehydrate were purchased from Sigma Aldrich (St. Louis, MO, USA) and were used as received. Silver nitrate (Honeywell, Seelze, Germany), hydroxylamine hydrochloride (NH_4_OH.HCl, Chemical Company SA, Iasi, Romania), ninhydrin monohydrate (VWR Life Science, Solon, OH, USA), and ascorbic acid (Scharlab S.L., Barcelona, Spain) were analytical-grade chemicals, used without purification.

### 4.2. Synthesis of Oxidized Gum Arabic (OGA)

OGA was obtained by the oxidation reaction of GA using the method proposed by Bruneel and Schacht [91] with slight modifications. In brief, gum arabic in aqueous solution was oxidized using potassium periodate (10:4 molar ratio between GA structural unit and KIO_4_) under mild stirring for 6 h in the dark at room temperature (RT). After this time, ethylene glycol in a 1:1 molar ratio to KIO_4_ was added to deactivate the unreacted periodate. The resulting product was purified by dialysis (dialysis bag with a molecular weight cut off 10–12 kDa) for 48 h against distilled water and was recovered by freeze-drying.

### 4.3. Preparation of AgNPs Covered with OGA (OGA-AgNPs)

Firstly, 30 mL of OGA aqueous solution (10 g/L) was prepared and the pH was adjusted to 7 using NaOH. Then, 15 mL of 10 mM AgNO_3_ solution (1:5.86 molar ratio between Ag and −CHO groups in OGA) was added, and the mixture was maintained at 60 °C on a water bath under magnetic stirring (1000 rpm). After 20 h, the obtained brown solution was dialyzed against water to remove the residual Ag and NO_3_ ions for 3 days, changing the water every 24 h; then, the OGA-AgNPs were recovered by freeze-drying.

### 4.4. Preparation of Hydrogels

The gelatin and OGA solutions, with concentrations of 10 wt.% each, were prepared using phosphate buffer (PB) with pH = 7.4 at 37 °C. Afterwards, at the same temperature, warm OGA solution was added over the gelatin solution under mild stirring. After stirring for two minutes (to ensure homogenization avoiding the gelation due to the fast cross-linking between OGA and gelatin), the mixtures were cast into Petri dishes (30 mm in diameter) and left for 20 h at 37 °C in the oven to ensure the chemical cross-linking of the hydrogels. Finally, the Gel/OGA hydrogels were dried for 48 h at −57 °C and 0.045 mbar using an Alpha 1-2 LD Martin Christ freeze-dryer (Osterode am Harz, Germany). To remove the macromolecules that were not involved in the chemical reaction, the hydrogels were washed with distilled water at 37 °C, for 2 days, then lyophilized.

The composite hydrogels loaded with different amounts of AgNPs (Gel/OGA-AgNPs) were obtained by partially or entirely replacing the OGA solution (10 wt% concentration) with OGA-AgNPs (10 wt%) solution. The following steps were the same as described above.

### 4.5. Characterization of OGA and OGA-AgNPs

The *content of the aldehyde groups* from OGA and OGA-AgNPs was determined using hydroxylamine hydrochloride method [92]. Briefly, 0.1 g sample was dissolved in 25 mL of NH_4_OH·HCl solution (0.25 M) with pH 4. After 20 h, the HCl released during the reaction between aldehyde groups and hydroxylamine hydrochloride was potentiometrically titrated with NaOH 0.1 M.

The intrinsic viscosities ([η]) of GA and OGA were determined in 1 N NaOH using an Ubbelohde viscometer at 25 °C. To avoid the formation of intermolecular hemiacetal linkages that can influence the molecular mass, the aldehyde groups from OGA were reduced after synthesis with sodium borohydride [91]. The *molecular weight* ($M_{w}$) was obtained from the Mark–Houwink equation ($\left[\eta \right]=K{\cdot M}_{w}^{a}$), using the values of *K* and a from the literature: $K=1.3\times 10^{-2}$, a=0.54 [93].

The *FTIR spectra* of the GA, OGA, and OGA-AgNPs samples were performed in KBr pallets, using a VERTEX 70 FT-IR spectrometer (Bruker, Ettlingen, Germany).

The *optical properties* of OGA-AgNPs were analyzed from the UV–Vis spectra of the aqueous solutions recorded on an Evolution 201 UV–Visible Spectrometer (Thermo Fisher Scientific, Waltham, MA, USA). At different time intervals, 0.1 mL was taken from the OGA-AgNPs solutions and diluted to 3 mL before recording the UV–Vis spectra.

The *dimensional analysis* of OGA-AgNPs in solution and their *zeta potential* at 25 °C were determined by Dynamic Light Scattering (DLS) using a ZetasizerNano ZS (Malvern Zetasizer NS-Malvern Instruments, Malvern, UK).

The *silver content* in OGA-AgNPs was determined by atomic absorption spectroscopy (AAS). The nanoparticles were dispersed in 10% HNO_3_, and then diluted with distilled water to a known volume. The AAS measurements were carried out in air/acetylene flame using a ContrAA 800 spectrometer (Analytik Jena, Jena, Germany).

To study the *morphology* of the OGA-AgNPs, an aqueous suspension of the sample (1 mg/mL) was applied to a 300-mesh copper grid coated with carbon and analyzed by transmission electron microscopy (TEM) using a Hitachi High-Tech HT7700 microscope (Tokyo, Japan) operating in “high contrast” mode. The nanoparticles’ diameters were measured using ImageJ 1.52 software.

### 4.6. Physicochemical Characterization of Hydrogels

The *gel fraction* (*GF*, %) was calculated with Equation (1) using the weights of the hydrogels after the first freeze-drying ($W_{1}$) and after purification ($W_{2}$):

(1) $$GF=\frac{W_{2}}{W_{1}}\times 100$$

The purification was performed in distilled water for two days at 37 °C to solubilize and remove the unreacted gelatin. Therefore, GF can be considered an indirect measure of the chemical cross-linking of the polymers (Gel, OGA, and OGA-AgNPs).

The *content of free amino groups* before and after cross-linking of gelatin was determined by ninhydrin assay [94,95]. In this respect, 8 mg hydrogel (without purification) was swollen in 1 mL of water, then 1 mL ninhydrin reactive was added, and the vial was kept for 30 min at 100 °C in a boiling water bath. The absorbance at 570 nm was measured after cooling and dilution (1:20) with a water/alcohol mixture (50:50 vol). The cross-linking degree was calculated by comparing the determined content of free amino groups in the hydrogels with the theoretical value of amino groups introduced by the gelatin (un-cross-linked).

The *porosity* of the dried hydrogels was determined by the liquid displacement method [96,97] using anhydrous ethanol as the non-wetting solvent. The size of the freeze-dried samples was initially measured to calculate the volume (V, cm^3^). The samples were weighed (*W*_0_) and immersed in ethanol at room temperature; after 5 min, they were withdrawn and the saturated weight of the hydrogel was measured (*W_s_*). The porosity (*P*, %) was calculated using Equation (2):

(2) $$P\;(\%)=\frac{\left(W_{s}-W_{0}\right)}{\rho V}\times 100$$

where ρ is the density of ethanol (0.785 g/cm^3^).

The *swelling behavior* of the hydrogels was studied at 37 °C in simulated physiological conditions of healthy skin (phosphate buffer (PB) pH = 5.5) and infected wounds (PB, pH = 7.4) [50] by the gravimetric method. The swelling ratio (*SR*) was calculated using Equation (3):

(3) $$SR=\frac{\left(W_{w}-W_{d}\right)}{W_{d}}\times 100$$

where $W_{d}$ is the weight of the dried sample and $W_{w}$ is the weight of the wet sample.

The *morphology and the elemental composition* of the freeze-dried hydrogels were evaluated using a Verios G4 UC Scanning Electron Microscope (Thermo Fisher Scientific, Brno, Czech Republic) equipped with an Octane Elite SDDs EDX detector (Ametek, Berwyn, PA, USA). The hydrogels were sectioned and coated with platinum. SEM investigations were performed in high-vacuum mode using a secondary electron detector at an accelerating voltage of 5.0 kV.

The *silver content* in the composite hydrogels was also determined by *AAS*. The hydrogel samples (10 mg) were first digested with 65% HNO_3_ (0.3 mL) and then diluted with distilled water to obtain the silver concentration in the calibration range (2–8 mg/L).

The *release of silver* from the composite Gel/OGA-AgNPs hydrogels was studied by immersion of approx. 12 mg sample in 5 mL of PB, pH = 7.4, at 37 °C for different time periods (6 h, and 1, 2, 4, and 7 days). After the established time, the solution was extracted, acidified with HNO_3_ 65%, and the metal concentration in solution was determined by AAS.

### 4.7. Mechanical Properties

Uniaxial compression tests were performed using a Brookfield Texture PRO CT3^®^, Texture Analyzer (Brookfield Engineering Laboratories Inc., Middleborough, MA, USA). The hydrogel samples (cylinders measuring 15 mm diameter and 9 mm height) were freeze-dried, washed with distilled water for 20 h to remove the uncross-linked polymers, and then immersed in PB (pH = 5.5) for 4 h. The compression tests were performed at room temperature with a 0.2 mm/s deformation speed. The compressive stress (σ) was calculated as σ=F/A, where F is the force applied (N), and A is the cross-sectional area of the hydrogel (m^2^); meanwhile, the strain (ε) was calculated as $\varepsilon =\Delta l/l_{0}$, where Δl is the change in length, and $l_{0}$ is the initial length. The compression elastic modulus (E=σ/ε) was obtained as the slope of the initial linear part of the stress–strain curve (ε=0.06−0.16). The ultimate compression strength was the maximum compression stress applied to the hydrogel before the failure.

### 4.8. Hydrolytic Degradation

The hydrolytic degradation of the hydrogels in physiological conditions was investigated by the gravimetric method. The dried hydrogel samples (around 80 mg) were weighed and then immersed in PB with pH = 5.5 (80 mL). After 2 or 4 weeks at 37 °C in an oven, the hydrogels were withdrawn from the buffer, washed with water for 6 h to remove the phosphate ions, and then dried by lyophilization and weighed. The mass loss (%) was calculated as:

(4) $$Mass\;loss(\%)=\frac{W_{1}-W_{2}}{W_{1}}\times 100$$

where $W_{1}$ is the initial weight of the hydrogel, and $W_{2}$ is the weight of the dried hydrogel after incubation.

### 4.9. Antioxidant Activity

The radical scavenging activity in aqueous solution was determined using the 2,2′-Azino-bis-(3-ethyl)benzothiazoline-6-sulfonic acid (ABTS) assay, where the ABTS^●+^ radical cations were generated by the oxidation of ABTS with potassium persulfate according to Re et al. [98], and the reduction in these radicals was measured by UV–Vis spectroscopy. For this assay, ABTS solution (7 mM) was mixed with 2.5 mM K_2_S_2_O_8_ solution and, after 16 h in the dark, the mixture with the formed radicals was diluted with water to obtain an absorbance value around 0.7. To measure the antioxidant activity of gelatin, OGA, and OGA-AgNPs, 0.2 mL of the sample solutions (0.4–2 mg/mL) was mixed with 2 mL of ABTS^●+^ solution and the absorbance at 732 nm was measured after 2 h. The ABTS^●+^ scavenging activity was calculated as:

(5) $${\mathrm{ABTS}}^{●+}\;\mathrm{scavenging}\;\mathrm{ability}\;(\%)=\frac{A_{c}-A_{s}}{A_{c}}\times 100$$

where $A_{c}$ is the absorbance of the control (2 mL of ABTS^●+^ with 0.2 mL water in this case) and $A_{s}$ is the absorbance of the sample. The ascorbic acid standard curve was also obtained using solutions with different concentrations of ascorbic acid (30–450 µmol/L).

The scavenging activity of the composite hydrogels was measured by adding 15 mg of dried sample into 3 mL ABTS^●+^ solution. The decrease in the absorbance was followed over time.

### 4.10. Antibacterial Activity

The minimum inhibitory concentration (MIC) of OGA-AgNPs was determined by the broth dilution method performed in 96-well microtiter plates. Bacterial culture grown to log phase was adjusted to 1 × 10^8^ cells/mL in Mueller–Hinton (MH) broth and then inoculants of 50 μL were mixed with 50 μL of serial dilutions of samples and afterward incubated at 37 °C for 24 h. The antibacterial activity was determined based on turbidity measured using a FLUOstar^®^ Omega microplate reader (BMG LABTECH, Ortenberg, Germany). The experiments were performed in triplicate.

The antibacterial activity of the hydrogel samples was performed using a viable cell-counting method [54]. Two reference bacterial strains were used, represented by *Staphylococcus aureus* ATCC25923 and *Escherichia coli* ATCC25922. These were refreshed on nutrient agar (NA) at 37 °C. Microbial suspensions were prepared with these cultures in a sterile nutrient broth medium to obtain turbidity that was optically comparable to that of 0.5 McFarland standards. A measure of 20 mg of the hydrogels was placed into a solution that contained 0.5 mL of the bacterium suspensions and 4.5 mL 1×PBS (phosphate-buffered saline 7.4, Gibco, Thermo Fisher Scientific, Paisley, UK) and incubated for up to 24 h in a shaker at 37 °C. A control experiment was also conducted. A measure of 1 µL of the control samples and of the treated samples was removed at determined periods of incubation time and spread on Plate Count Agar (PCA) plates. The number of colonies was counted after 24 h of incubation at 37 °C. All tests were carried out in triplicate to verify the results. After incubation, the plates were analyzed with SCAN1200^®^, version 8.6.10.0 (Interscience, Saint Nom la Bretêche, France), and the number of colonies was expressed as the mean ± standard deviation (SD) performed with GraphPad Prism software version 7.00 for Windows (GraphPad Software, La Jolla, CA, USA, www.graphpad.com). The inhibition ratio of the growth of bacteria (IRG) was calculated with the following formula:

(6) $$IRG\left(\%\right)=\frac{\left(Na-Nb\right)}{Na}\times 100$$

where N*a* and N*b* are the average values of colonies of the control group and the experimental groups, respectively.

### 4.11. Statistical Analysis

The results were expressed in mean standard deviation (S.D.). Statistical differences (*p* < 0.05) between groups were analyzed using one-way ANOVA followed by *t*-test Tukey’s post hoc test.

## Figures and Tables

**Table 1:** Influence of the ratio between Gel and OGA and of the AgNPs addition on the properties of the hydrogels.

Sample Code	Initial Mixture			Hydrogel Characteristics					Ag (wt.%) **
	Gel:OGA: OGA-AgNPs (%, wt:wt:wt)	CH=O: NH_2_ Molar Ratio	Ag (wt. %)	GF (%)	Cross-Linking of NH_2_ Groups (%)	Porosity (%)	SR * (g/g)		
							pH = 5.5	pH = 7.4	
H1	60:40:0	5.7:1	-	85.6 ± 0.4	55.9 ± 3.6	70.7 ± 0.2	7.2 ± 0.4	7.8 ± 0.2	-
H2	50:50:0	8.6:1	-	86.6 ± 0.6	67.6 ± 1.1	76.2 ± 1.0	8.6 ± 0.3	8.6 ± 0.1	-
H3	40:60:0	12.9:1	-	87.5 ± 0.1	70.0 ± 1.4	77.3 ± 0.3	9.1 ± 0.4	8.9 ± 0.3	-
N1	40:30:30	10.8:1	0.9	87.3 ± 0.3	63.7 ± 2.8	72.4 ± 1.0	8.6 ± 0.3	8.4 ± 0.2	0.61 ± 0.02
N2	40:0:60	8.7:1	1.8	87.4 ± 0.5	58.9 ± 1.1	64.6 ± 1.2	8.2 ± 0.2	8.0 ± 0.1	1.32 ± 0.08

* SR represents the swelling ratio determined with Equation (3); ** determined by AAS.

## Data Availability

The data presented in this study are available on request from the corresponding authors.

## Supplementary Materials

The following supporting information can be downloaded at: https://www.mdpi.com/article/10.3390/gels11060429/s1, Figure S1: ^1^H-NMR spectra of gum arabic (GA) and oxidized gum arabic (OGA) in D_2_O; Figure S2: FT-IR spectra of gelatin (Gel), OGA-AgNPs and composite Gel/OGA-AgNPs hydrogel (N2) and deconvoluted FT-IR spectrum (800–1900 cm^−1^) of N2 sample (inset); Figure S3: Release profiles of Ag from composite Gel/OGA-AgNPs hydrogels in phosphate buffer pH = 7.4 and 37 °C.
