# Supplementary material for: One-Pot Synthesis of Gelatin/Gum Arabic Hydrogels Embedding Silver Nanoparticles as Antibacterial Materials
Source: Gels. 2025 Jun 3;11(6):429. doi: 10.3390/gels11060429 (PMC12191848; doi:10.3390/gels11060429)
Supplement: Supplementary file 1 [file gels-11-00429-s001.zip › gels-3647425-supplementary.pdf]

Electronic Supplementary information (ESI)

One-pot synthesis of Gelatin/Gum Arabic hydrogels embedding silver nanoparticles as antibacterial materials

Irina Popescu, Irina Mihaela Pelin, Irina Rosca, and Marieta Constantin

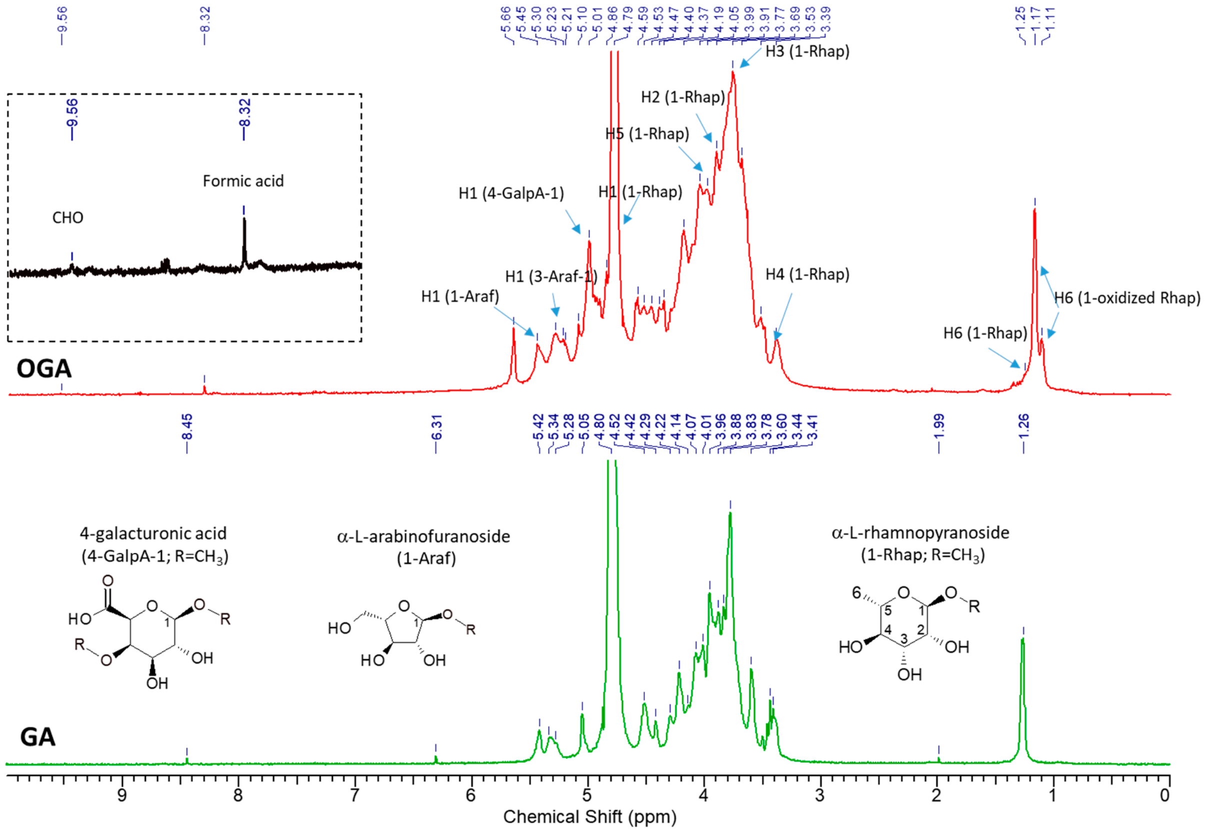

Figure S1. ^{1}H-NMR spectra of gum arabic (GA) and oxidized gum arabic (OGA) in D_2O.

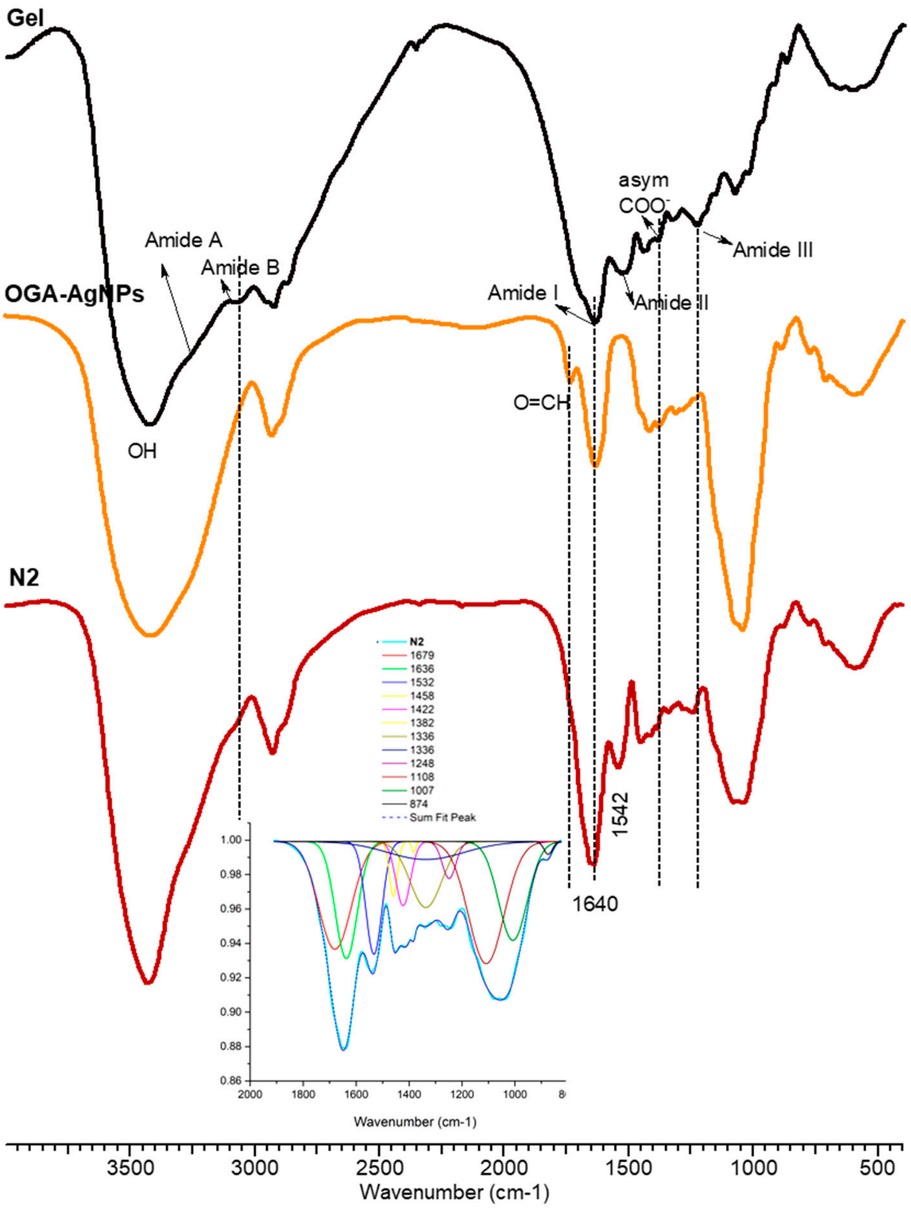

Figure S2. FT-IR spectra of gelatin (Gel), OGA-AgNPs and composite Gel/OGA-AgNPs hydrogel (N2) and deconvoluted FT-IR spectrum (800-1900 cm^{-1}) of N2 sample (inset).

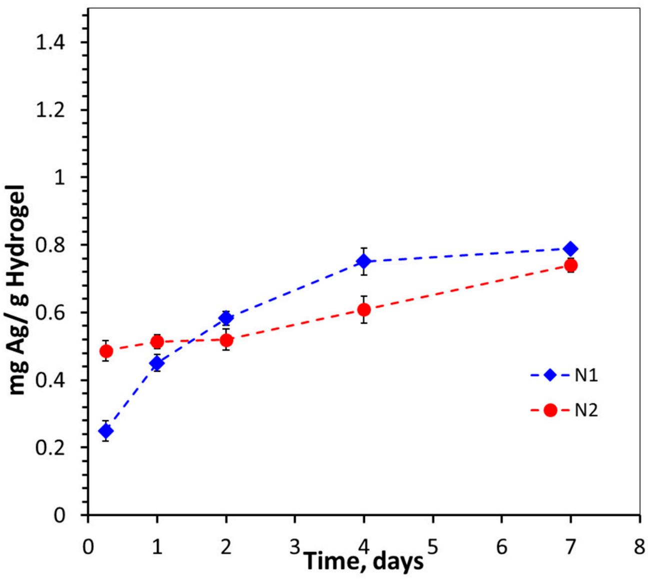

Figure S3. Release profiles of Ag from composite Gel/OGA-AgNPs hydrogels in phosphate buffer pH = 7.4 and 37 °C.
